# Traité international de Psychologie pathologique

**Published:** 1910-06

**Authors:** 


**Traité international de Psychologie pathologique.** Edited by Dr. A. MARIE and Collaborators. Tome I. Pp. vii, 1,028. Paris: FÉLIX ALCAN. 1910.

This book of over a thousand pages is a review of the scientific, social and philosophic aspects of morbid psychiatry, prepared by Dr. A. Marie and eminent collaborators of various nationalities. It is the first of three volumes, already in the press, needed to complete the scheme set forth; the other two will be issued by the end of the current year.

In the introductory chapters is a plea for the inclusion of morbid psychology as an integral part of neuro-biology, made by Dr. Grasset and endorsed by Dr. Greco, who also gives a critical and historical survey of the gradual development of psychiatric knowledge, the social relationships of morbid psychology in regard to marriage, education, hygiene and prophylaxis. Degenerescence and physiological experiment on animals are then treated of. Very complete articles follow, which deal with anthropology in relation to insanity; with studies in anatomy and function; chemistry of brain substance; the application of laboratory methods, metric and otherwise; clinical examination of patients; morbid anatomy and histology of brain; peripheral and visceral nervous systems; psychic evolution; backward children and criminal lunacy.

The length and complexity of the numerous essays renders any detailed notice of this volume impracticable. The names of its contributors constitute a sufficient guarantee for the general excellence. Especially valuable is the well-illustrated article on lesions of the cortical nerve cells, by Marinesco. An issue in English of this work would be welcome, but even failing this, it should be in the hands of all workers in morbid psychology. Dr. Marie is to be congratulated on this instalment of his scheme. It is well produced.

